# Circular RNAs as Targets for Developing Anticancer Therapeutics

**DOI:** 10.3390/cells14141106

**Published:** 2025-07-18

**Authors:** Jaewhoon Jeoung, Wonho Kim, Hyein Jo, Dooil Jeoung

**Affiliations:** Department of Biochemistry, Kangwon National University, Chuncheon 24341, Republic of Korea; heyjhw@kangwon.ac.kr (J.J.); kimwonho99@kangwon.ac.kr (W.K.); qnfdudn1212@gmail.com (H.J.)

**Keywords:** anticancer drug resistance, cancer diagnosis, circular RNAs, circular RNA synthesis, circular RNA vaccine, clinical trials, immune responses

## Abstract

Circular RNA (CircRNA) is a single-stranded RNA arising from back splicing. CircRNAs interact with mRNA, miRNA, and proteins. These interactions regulate various life processes, including transcription, translation, cancer progression, anticancer drug resistance, and metabolism. Due to a lack of cap and poly(A) tails, circRNAs show exceptional stability and resistance to RNase degradation. CircRNAs exhibit dysregulated expression patterns in various cancers and influence cancer progression. Stability and regulatory roles in cancer progression make circRNAs reliable biomarkers and targets for the development of anticancer therapeutics. The dysregulated expression of circRNAs is associated with resistance to anticancer drugs. Enhanced glycolysis by circRNAs leads to resistance to anticancer drugs. CircRNAs have been known to regulate the response to chemotherapy drugs and immune checkpoint inhibitors. Exogenous circRNAs can encode antigens that can induce both innate and adaptive immunity. CircRNA vaccines on lipid nanoparticles have been shown to enhance the sensitivity of cancer patients to immune checkpoint inhibitors. In this review, we summarize the roles and mechanisms of circRNAs in anticancer drug resistance and glycolysis. This review discusses clinical applications of circRNA vaccines to overcome anticancer drug resistance and enhance the efficacy of immune checkpoint inhibitors. The advantages and disadvantages of circRNA vaccines are also discussed. Overall, this review stresses the potential value of circRNAs as new therapeutic targets and diagnostic/prognostic biomarkers for cancer

## 1. Biogenesis of CircRNAs

**Figure 1:**
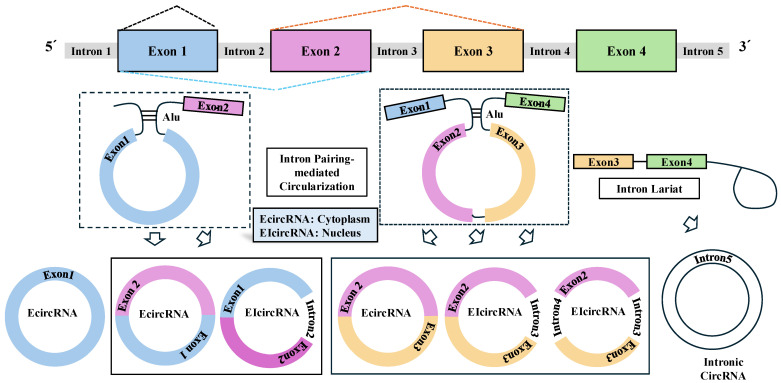
Biogenesis of circRNAs. Back splicing of exons leads to the formation of circular RNAs with a 3′,5′-phosphodiester bond at the back-splicing junction site. The efficiency of back splicing is usually low, resulting in low level of circRNA. EcircRNAs, containing only exons, are mainly located in the cytoplasm and regulate various cellular functions. EIciRNAs and ciRNAs act in the nucleus. Hollow arrows denote the direction of reaction.

Circular RNA (circRNA) is a class of natural or synthetic closed single-stranded circular RNA. The formation of circRNAs occurs co-transcriptionally and post-transcriptionally. CircRNAs are divided into three categories: exonic circRNAs (EcircRNAs), circ intronic RNAs (ciRNAs), and exon–intron circRNAs (EIciRNAs) [1] (Figure 1). CircRNAs are found not only in mammalian cells but in various eukaryotes [2,3,4,5]. More than 10% of the genes expressed in mammalian cells can produce circRNAs [6]. Exogenous and endogenous circRNAs show tissue- and cell-type-specific expression pattens [7].

CircRNAs, transcribed by RNA polymerase II in eukaryotic cells from precursor mRNA (pre-mRNA) molecules, are formed via back splicing [8] (Figure 1). CircRNAs lack the 5′-cap and 3′-polyA tail structures [9,10]. The lack of 5′ cap and a 3′ poly(A) tail leads to resistance to degradation by RNA nucleases. CircRNAs were initially thought to be a byproduct of erroneous splicing. Back splicing involves the formation of a 3′,5′-phosphodiester bond between the 3′-end of an exon and the 5′-end of either its exon or an upstream exon [11]. The circularization of linear RNA requires RNA-binding proteins (RBPs) or Alu elements [12,13], which ensure the proximity of the regions that need to be circularized [14,15].

The efficiency of back splicing is much lower than that of canonical splicing, resulting in a generally low abundance of circRNAs [16]. CircRNAs arising from exons function in the cytoplasm. CircRNAs containing introns play regulatory roles in the nucleus. Exosomal circRNAs are found within the exosomes, mediate cellular interactions and play important roles in various cellular processes [7]. The wide distribution of exosomal circRNAs can make them valuable diagnostic and prognostic markers.

## 2. Regulation of Gene Expression by CircRNAs

**Figure 2:**
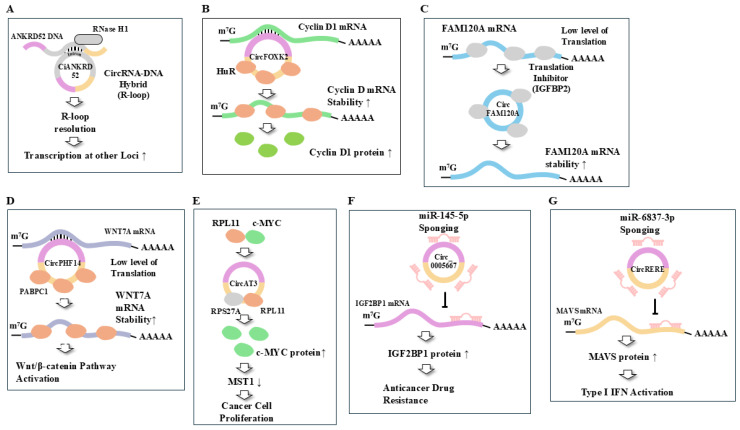
Cellular functions of circRNAs. (**A**) Intronic circ RNA, ciankrd52, recruits RNase H1 by forming R loop with its parental DNA. Resolution of R loop then leads to degradation of ciankrd52 and enhances transcription at other circRNA-producing loci. (**B**) Interaction with mRNA: Binding of circFOXK2 to cyclin D1 mRNA stabilizes cyclin D1 mRNA by recruiting ELAVL1/HuR. The increased level of cyclin D1 protein promotes breast cancer progression. (**C**) Effect on translation: Binding of circFAM120A to translation inhibitor enhances translation of FAM120A mRNA. (**D**) Effect on protein interactions (Scaffold): CircAT3 induces interactions involving RPS27A and RPL11, which inhibits the interaction between RPL11 and c-MYC. C-MYC decreases the expression of MST1 to cause cancer cell proliferation. (**E**) Effect on stability of mRNA: Interaction between circPHF14 and PABPC1 stabilizes WNT7A mRNA, which in turn activates the Wnt/β-catenin pathway to promote EMT. (**F**) Sponge miRNA: Circ_0005667 increases the expression of IGF2BP1 by sponging miR-145-5p, which results in anticancer drug resistance. (**G**) Activation of immune response: CircRERE activates type I IFN by sponging miR-6837-3p and increasing the expression of MAVS expression. T bar denotes the inhibition of reaction. Hollow arrows and arrows denote the direction of reaction. ↓ denotes the decreased expression/activity and ↑ denotes the increased expression/activity.

The identification of circRNAs has been made possible with the aid of high-throughput RNA sequencing (RNA-seq) and circRNA-specific bioinformatics algorithms in eukaryotes [17,18,19]. Thus, circRNA is now considered a general feature of gene expression. CircRNAs play critical roles in regulating gene expression at both the transcriptional and post-transcriptional levels [20]. Many circRNAs containing introns tend to form DNA: RNA hybrids known as R-loops, which affect DNA replication and transcription [20]; intronic circ RNA, ciankrd52, forms R loop, which, in turn, recruit RNase H1 to resolve R loop. Resolution of the R loop then leads to degradation of ciankrd52 and enhances transcription at other circRNA-producing loci [21] (Figure 2A). CircRNAs can also regulate gene expression through interactions with miRNAs, mRNAs, and RBPs, thereby regulating splicing, translation, cancer cell invasion, angiogenesis, and immune evasion [22,23,24,25,26,27,28].

CircRNAs can indirectly regulate the expression of downstream target genes of miRNAs by sponging functional miRNAs [29,30,31]. An elevated level of miR-661 is positively associated with malignant colorectal cancer phenotypes [32]. Circ_RUSC2 increases the expression of tumor-suppressor candidate 2 (TUSC2) by sequestering miR-661, which leads to the suppression of colorectal cancer progression [32]. The hsa_circ_0020303 induces clear cell renal cell carcinoma (ccRCC) progression by sponging miR-125a-5p, which promotes eukaryotic translation initiation factor 4E binding protein 1 (EIF4EBP1) expression [33]. CircRNAs can bind to linear mRNA to regulate tumor growth [34]. CircFOXK2 binds to cyclin D1 mRNA and recruits RNA-binding protein ELAVL1/HuR, which stabilizes cyclin D1 mRNA in ER-positive breast cancer cells [34] (Figure 2B). CircFOXK2 is overexpressed in non-small cell lung cancers (NSCLCs) [35]. CircFOXK2 binds to polyadenylate-binding protein 1 (PABPC1) and stabilizes stathmin (STMN1) mRNA [35].

CircFAM120A binds to translation inhibitor insulin-like growth factor binding 2 (IGFBP2) and prevents FAM120A mRNA from binding to the IGFBP2, which results in the high-level translation of FAM120A mRNA [36] (Figure 2C). CircCLASP2 increases the translation of protein-L-isoaspartate (D-aspartate) O-methyltransferase (PCMT1) through binding to nuclear DNA Helicase 9 (DHX9), which results in nasopharyngeal carcinoma (NPC) progression [37]. This pathway may serve as a potential therapeutic target for NPC. CircPAFAH1B2 and circUBAP2 bind to IGFBP2 and stabilize dickkopf 3(DKK3) and aryl hydrocarbon receptor (AHR) mRNAs to promote the peritoneal dissemination of ovarian cancer [38]. These reports suggest that circRNAs can regulate various biological processes by regulating transcription and translational processes.

## 3. CircRNAs and RNA-Binding Proteins

CircRNAs can interact with proteins, acting as a protein sponge [39,40]. CircRNAs can interact with RNA-binding proteins (RBPs) and regulate transcription, translation, signaling pathways, tumor growth and metastasis, immune responses, and drug resistance [41,42,43,44,45,46,47,48,49].

CircPHF14 binds to PABPC1 and stabilizes WNT7A mRNA, which activates the Wnt/β-catenin pathway to promote the metastasis of pancreatic ductal adenocarcinoma (PDAC) [47] (Figure 2D). CircAT3 can act as scaffolds to induce interactions between ribosomal protein S27a (RPS27A) and ribosomal protein L11 (RPL11) [50] (Figure 2E). This interaction inhibits the interaction between RPL11 and c-MYC, which in turn leads to c-MYC suppression of macrophage-stimulating 1 (MST1) expression [50] (Figure 2E). The decreased expression of MST1 promotes prostate cancer cell proliferation [50]. CircKIAA1797 inhibits cuproptosis by inhibiting ferredoxin 1 (FDX1) expression and promotes lung cancer development [51]. CircKIAA1797 binds to the signal transducer and activator of transcription 1 (STAT1) protein to inhibit the expression of lipoyltransferase 1 (LIPT1) in lung cancer cells [51].

CircMETTL6 is downregulated in ovarian cancer, and the overexpression of circMETTL6 inhibits tumor growth and metastasis [52]. CircMETTL6 binds to non-POU domain containing octamer-binding protein (NONO) and inhibits the binding of NONO to RNA polymerase II subunit A (POLR2A), which results in the suppression of ovarian cancer progression [52].

CircRNAs can recruit RBPs into specific locations. For example, circMYH9 recruits EIF4A3 to increase the expression of sperm-associated antigen 6 (SPAG6) expression, which promotes breast cancer cell proliferation by activating the PI3K/AKT signaling pathway [53].

CircCCNY binds to HSP60 and induces the degradation of HSP60 through its interaction with the E3 ubiquitin ligase SMURF1 [54]. CircCCNY can enhance CD8^+^ T-cell infiltration and suppress immune evasion by inhibiting the MAPK/c-Myc/programmed death ligand-1 (PD-L1) signaling pathway in hepatocellular carcinoma (HCC) cells [54]. This suggests the role of circRNAs in the immune evasion of cancer cells.

CircEMSY induces the aggregation of the RNA-binding protein TAR DNA-binding protein 43 (TDP-43), which in turn leads to the stimulation of the cGAS-STING pathway for the activation of the antiviral immune response [55].

## 4. Roles of CircRNAs in Chemotherapy Resistance

Diverse variants and high stability can make circRNAs regulators of tumorigenesis. The circRNAs can bind to and inhibit the function of miRNAs, which can alter the expression of target genes involved in cancer initiation and/or progression [56]. Thus, circRNAs can regulate the responses to anticancer drugs. Dysregulated circRNA expression profiles contribute to tumorigenesis and anticancer drug resistance [57,58,59,60,61]. The expression level of Circ_0000376 is upregulated in PTX-resistant NSCLCs [59]. Circ_0000376 confers resistance to PTX in NSCLCs by sponging miR-1298-5p and by increasing the expression of karyopherin subunit alpha 4 (KPNA4) [59]. Circ_0076305 confers resistance to paclitaxel (PTX) in non-small cell lung cancer cells (NSCLCs) by sponging miR-936 and increasing transmembrane serine protease 4 (TMPRSS4) expression [62]. CircQKI, upregulated in docetaxel (DTX)-resistant prostate cancer cells, promotes DTX resistance by sponging miR-188-3p and increasing the expression of Beclin-1 [63]. This indicates that resistance to DTX involves the activation of autophagic processes. Circ_0058608 is increased in NSCLC tissues and taxol-resistant NSCLC cells [64]. Circ_0058608 sponges miR-1299 and increases the expression of guanylate-binding protein 1 (GBP1) to promote taxol resistance of NSCLC [64]. Circ_0044556 functions as a competing endogenous RNA (ceRNA) for miR-665 and confers resistance to PTX in malignant breast cancer cells [65].

Circ_0005667 sponges miR-145-5p and enhances the resistance of endometrial cancer cells to cisplatin by increasing insulin-like growth factor 2 binding protein 1 (IGF2BP1) expression [66] (Figure 2F). Hsa_circ_0006006, circRNF13, and circKRT75 confer cisplatin resistance across different cancer types [67,68,69]. CircKIAA0182 promotes cisplatin resistance and tumor progression in NSCLC through interaction with the RNA-binding protein YBX1 [70]. Overexpression of circABCC4 (hsa_circ_0030582) confers resistance to oxaliplatin and is positively correlated with shorter progression-free survival (PFS) in late-stage patients with pancreatic ductal adenocarcinoma [71].

The expression of circMYBL1 (has_circ_0136924) is downregulated in osimertinib-resistant NSCLC cells [72]. The downregulation of circMYBL1 promotes the invasion of osimertinib-sensitive NSCLC cells [72]. Thus, circMYBL1 can function as a tumor suppressor.

**Figure 3:**
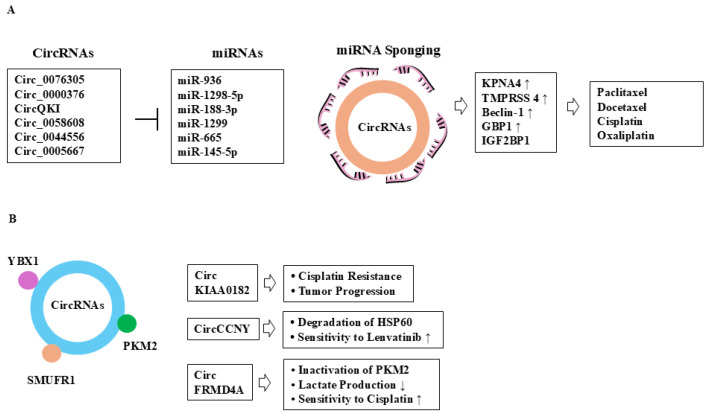
Roles of circRNAs in chemotherapy resistance. CircRNAs can sponge miRNAs (**A**) and proteins (**B**) to confer resistance to chemotherapy drugs. T bar denotes the inhibition of reaction. Hollow arrows denote the direction of reaction. ↓ denotes the decreased expression/activity and ↑ denotes the increased expression/activity.

CircFRMD4A, induced by p53, inactivates the pyruvate kinase PKM2, leading to a decrease in lactate production [73]. The downregulation of PKM2 enhances the sensitivity of esophageal squamous cell carcinoma to cisplatin by regulating autophagy [74]. The inhibition of PKM2 decreases the expression of PD-L1 and suppresses bladder cancer growth and enhances antitumor immune responses [75]. These reports suggest that targeting the glycolytic pathway may enhance the sensitivity to anticancer therapeutics. CircCCNY can also enhance the sensitivity of HCC cells to lenvatinib [54] by inducing the degradation of HSP60. In doing so, circCCNY interacts with E3 ubiquitin ligase SMURF1 to induce the degradation of HSP60 [54]. In other words, circCCNY acts as a scaffold to regulate the response to anticancer drugs. Thus, circCCNY can be a potential biomarker for predicting sensitivity to lenvatinib. Table 1 summarizes the roles of circRNAs in resistance to chemotherapy. Figure 3 shows the mechanism of circRNA-promoted chemotherapy resistance.

## 5. Roles of CircRNAs in Immune Checkpoint Inhibitor Resistance

Programmed death ligand-l (PD-L1) interacts with programmed death 1 (PD-1) to mediate immune evasion by cancer cells [76]. Immune checkpoint inhibitors (ICIs), such as anti-PD-1/PD-L1 antibodies, have shown efficacy across various cancers. However, the primary and acquired resistance to ICIs makes the clinical application of ICIs challenging. CircRNAs regulate the expression of immune checkpoints in shaping the tumor immune microenvironment. Thus, immune evasion serves as the theoretical foundation for tumor immunotherapy.

CircRNAs modulate anticancer immunotherapy resistance by sponging miRNAs or serving as protein scaffolds. CircPETH-147aa impairs anticancer immunity by increasing RNA-binding protein HuR-dependent solute carrier family 43-member 2 (SLC43A2) mRNA stability and causing methionine deficiency in cytotoxic CD8^+^ T cells [77]. In other words, circPETH-147aa can promote immune evasion by inducing the apoptosis of cytotoxic CD8^+^ T cells. The inhibition of circPETH-147aa enhances the efficacy of anti-PD-1 antibody [77]. Circ-CPA4 sponges let-7 miRNA and increases the expression of PD-L1 expression to cause immune evasion in NSCLC, while the downregulation of circ-CPA4 promotes the activation of CD8^+^T cells [78]. By sponging miR-155-5p and miR-194-5p, circ_CHST15 upregulates PD-L1 expression, suppresses CD8^+^ T-cell activity, and induces CD8^+^ T-cell apoptosis to promote immune evasion [79]. The circ_0001006 is overexpressed in NSCLC tissues, and the downregulation of circ_0001006 leads to the activation of CD8^+^ T cells [80]. CircENTPD7 promotes immune evasion and metastasis of NSCLC cells by upregulating IGF2BP/PD-L1 axis [81].

A high level of circFAM64A(3) is positively associated with poor prognosis of bladder cancer [82]. CircFAM64A(3) promotes bladder cancer cell proliferation and immune evasion by sponging miR-149-5p and increasing the expression of PD-L1 [82]. CircAATF is upregulated in gall bladder carcinoma and promotes gall bladder cancer cell growth [83]. CircAATF increases the expression of PD-L1 by activating AKT and acting as a sponge for miR-142-5p [83]. CircAATF enhances the effect of anti-PD-L1 immunotherapy in a mouse model [83]. CircMGA enhances the infiltration of CD8^+^ T cells in bladder cancer by increasing the expression of CCL5 through interaction with heterogeneous nuclear ribonucleoprotein L (HNRNPL) [46]. Thus, the combination of circMGA and anti-PD-L1 or anti-PD-1 may suppress the growth of bladder cancer.

The expression levels of PD-L1 and interferon gamma receptor 2 (IFNGR2) are increased in nasopharyngeal carcinoma (NPC) [84]. Oncogenic circ RNA, circ_001377 prevents miR-498-3p from targeting IFNGR2 in NPC [84]. Thus, circ_001377/miR-498-3p interaction can confer resistance to anti-PD-L1 immune therapy.

CircSCUBE3 is highly expressed in human GC tissues and can predict poor prognosis in gastric cancer patients [85]. CircSCUBE3 inhibits the effect of anti-PD-L1 by binding to miR-744-5p in gastric cancer cells [85]. CircDLG1 is upregulated in metastatic gastric cancer (GC) tissues resistant to anti-PD-1 therapy [86]. A high level of circDLG1 is positively associated with poor prognosis in gastric cancer patients treated with anti-PD-1 therapy [86]. CircDLG1 sponges miR-141-3p and increases the expression of CXCL12, which promotes resistance to anti-PD-1 therapy [86].

CircPHLPP2 is upregulated in colorectal cancer patients with resistance to anti-PD-1 therapy [87]. The interaction between circPHLPP2 and interleukin enhancer-binding factor 3 (ILF3) facilitates the nuclear accumulation of ILF3, which impairs NK cell functions [87]. The downregulation of circPHLPP2 enhances the efficacy of anti-PD-1 in vivo [87]. CircNCOA3 is overexpressed in colorectal cancer patients with resistance to anti-PD-1 therapy [88]. The downregulation of circNCOA3 increases the number of CD8^+^ T cells while decreasing the number of myeloid-derived suppressor cells (MDSCs) [88]. The downregulation of circNCOA3 enhances the efficacy of anti-PD-1 treatment in mouse tumor models [88]. The overexpression of circRERE inhibits the malignant behaviors of colorectal cancer [89]. CircRERE sponges miR-6837-3p to upregulate mitochondrial antiviral signaling protein (MAVS) expression, which activates type I IFN signaling and antitumor immunity [89] (Figure 2G). The combination of the circRERE-AAV and anti-PD-1 antibody synergistically reduces tumor growth in preclinical models of colorectal cancer [89].

A high level of circFGFR4 is positively correlated with decreased CD8^+^ T-cell infiltration in breast cancer tissues and resistance to anti-PD-1 immunotherapy in triple-negative breast cancer (TNBC) patients [90]. CircFGFR4 prevents miR-185-5p from decreasing the expression of C-X-C motif chemokine receptor 4 (CXCR4) in breast cancer cells [90]. Thus, circFGFR4 can confer resistance to anti-PD-1 by regulating the miR-185-5p/CXCR4 axis. CircCFL1 enhances the interaction between HDAC1 and c-Myc, which promotes the stability of c-Myc via the inhibition of K48-linked ubiquitylation in breast cancer cells [91]. C-Myc enhances the expression of mutant p53, which promotes the stemness of TNBC cells [91]. CircCFL1 can enhance the immune evasion of TNBC cells by promoting the expression of PD-L1 and suppressing the antitumor immunity of CD8^+^ T cells [91].

Galectin-9 interacts with immune checkpoint molecules TIM-3 and PD-1 and enhances the function of CD8^+^ T cells [92]. CircE7 suppresses the function of T cells by decreasing the expression of galectin-9 in head and neck squamous cell carcinoma (HNSCC) [92]. CircE7 binds to acetyl-CoA carboxylase 1 (ACC1) and activates ACC1 [92]. Activated ACC1 decreases the expression of galectin-9 [92]. Thus, the combination of sicircE7 and anti-PD-1 may enhance the sensitivity to anti-PD-1 treatment.

CircNF1 contributes to esophageal squamous cell carcinoma (ESCC) malignant phenotypes and regulates CD8^+^ T-cell-mediated antitumor immunity. CircNF1 promotes the activation of the JAK-STAT3 pathway, which in turn increases the expression of PD-L1 [93]. CircNF1 interacts with annexin A1 (ANXA1), thereby increasing interaction between ubiquitin-specific protease 7 (USP7) and PD-L1 and enhancing PD-L1 stability [93]. Thus, the high level of circNF1 can confer resistance to anti-PD-L1 therapy.

CircRHBDD1 is highly expressed in GC tissues [94]. CircRHBDD1 increases the expression of PD-L1 and inhibits the infiltration of CD8^+^ T cells [94]. CircRHBDD1 binds to IGF2BP2, which results in the increased expression of PD-L1 by disrupting interaction between E3 ligase TRIM25 and IGF2BP2 [94]. This suggests that the combination of sicircRHBDD1 and anti-PD-L1 can overcome resistance immune checkpoint inhibitors.

CircSLCO1B3 is highly expressed in intrahepatic cholangiocarcinoma (ICC) tissues and positively correlated with lymphatic metastasis [95]. CircSLCO1B3 can also promote immune evasion via antagonizing PD-L1 degradation [95].

CircSOD2 is upregulated in HCC tissues and cells [96]. CircSOD2 increases the expression of annexin A 11 (ANXA11) by sponging miR-497-5p [96]. CircSOD2 promotes anti-PD-1 resistance via the miR-497-6p/ANXA11 axis [96].

Cytotoxic T-lymphocyte antigen 4 (CTLA-4), an immune checkpoint molecule, inhibits T-cell function. CTLA-4 inhibits the production of IL-2 and the proliferation of T cells [97]. The m^6^A-modified circQSOX1 sponges miR-326 and miR-330-5p to promote phosphoglycerate mutase 1 (PGAM1) expression, which promotes immune evasion by activating glycolysis [98]. Thus, targeting glycolysis can enhance the sensitivity to CTLA-4 inhibitors. The m^6^A-modified circQSOX1, recognized by IGF2BP2, can inhibit the response of the anti-CTLA-4 therapy response in patients with colorectal cancers (CRCs) [98]. Thus, the downregulation of circQSOX1 can overcome Treg cell-mediated immune therapy resistance in CRC.

**Figure 4:**
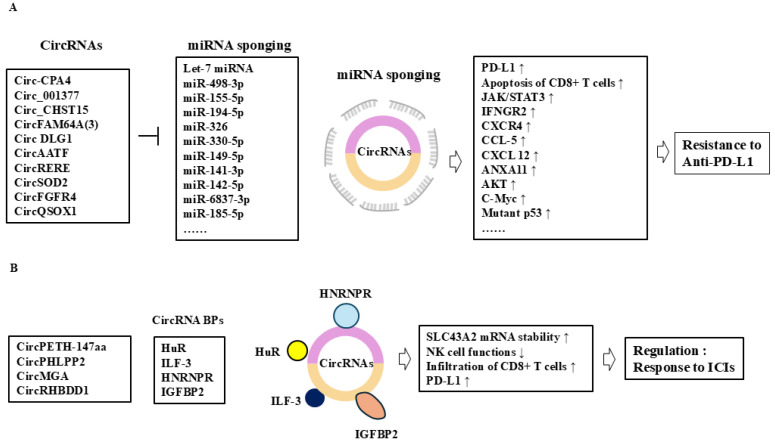
Roles of circRNAs in the responses to immune checkpoint inhibitors. CircRNAs can sponge miRNAs (**A**) and proteins (**B**) to regulate the responses to immune checkpoint inhibitors. T bar denotes the inhibition of reaction. Hollow arrows denote the direction of reaction. ↓ denotes the decreased expression/activity and ↑ denotes the increased expression/activity.

Exosomal circRNAs can participate in intercellular communication, affecting the progression of a variety of tumors [99]. Exosomal circCCAR1 sponges miR-125-5p, and the circCCAR1/miR-127-5p/WT1-associated protein (WTAP) feedback loop enhances the metastatic potential of HCC [100]. Exosomal circCCAR1 impairs the function of CD8^+^ T cells and promotes resistance to anti-PD-1 immunotherapy by stabilizing PD-1 [100]. Exosomal circWDR25 sponges miR-4474-3p and induces epithelial-to-mesenchymal transition (EMT) in HCC cells by regulating the expression of lipoxygenase (ALOX15) [101]. Exosomal circWDR25 also increases the expression of CTLA-4 and PD-L1 in HCC cells [101]. Thus, exosomal circWDR25 can transfer resistance to ICIs. Exosomal circUHRF1 sponges miR-449c-5p and increases the expression of T-cell immunoglobulin and mucin domain 3 (TIM-3), which leads to the inhibition of NK cell function [102]. These reports suggest that exosome-mediated cellular interactions may determine the response to immune checkpoint inhibitors. Thus, exosomal circRNAs can be potential targets for developing anticancer immune therapy. Table 2 summarizes the roles of circRNAs in immune evasion. Figure 4 shows the mechanisms of circRNA-promoted resistance to ICIs.

## 6. CircRNAs May Regulate the Responses to ICIs via Their Effects on Glycolysis

Metabolic reprogramming such as glycolysis contributes to cancer cell progression by remodeling the immunosuppressive tumor microenvironment (TME) [103]. This implies that circRNAs can regulate glycolysis. The overexpression of CircPVT1 increases the tumorigenicity of TNBC cell lines [4]. A high level of circPVT1 is positively associated with poor prognosis in breast cancer patients [4]. The downregulation of circPVT1 suppresses glycolysis by increasing the expression of miR-33a-5p [4].

CircRUNX1 promotes glycolysis and lactate generation, which results in the infiltration of regulatory T cell (Treg) by sponging miR-145 and increasing the expression of HK2 in NSCLC [56]. Thus, the circRUNX1/miR-145/HK2 axis can promote immune evasion by activating aerobic glycolysis.

CircZNF707 increases the expression of the muscle isoform of phosphofructokinase (PFKM) by sponging miR-668-3p and contributes to the progression of NSCLC [45]. The expression of circSEC24A is increased in NSCLC tissues, while that of miR-1253 is decreased [104]. CircSEC24A sponges miR-1253 and enhances the tumorigenic potential of NSCLCs and glycolysis [104]. CircST6GALNAC6 suppresses tumor growth by increasing parkin E3 ubiquitin ligase (PRKN) [105]. The overexpression of circST6GALNAC6 inhibits glycolysis and the proliferation of bladder cancer cells by promoting the degradation of hexokinase 1 (HK1) [105].

Circ_0043256 is upregulated in GC tissues [106]. The downregulation of circ_0043256 suppresses the tumorigenic potential of GC cells by increasing the expression of miR-593-5p and decreasing the expression of ribonucleoside-diphosphate reductase subunit M2 (RRM2) to inhibit the glycolytic pathway [106]. The expression of Hsa_circ_0001756 is increased in GC tissues [107]. Hsa_circ_0001756 increases the expression of phosphoglycerate kinase 1 (PGK1) through binding to polypyrimidine tract-binding protein 1 (PTBP1) and promotes glycolysis by sponging miR-185-3p [107].

**Figure 5:**
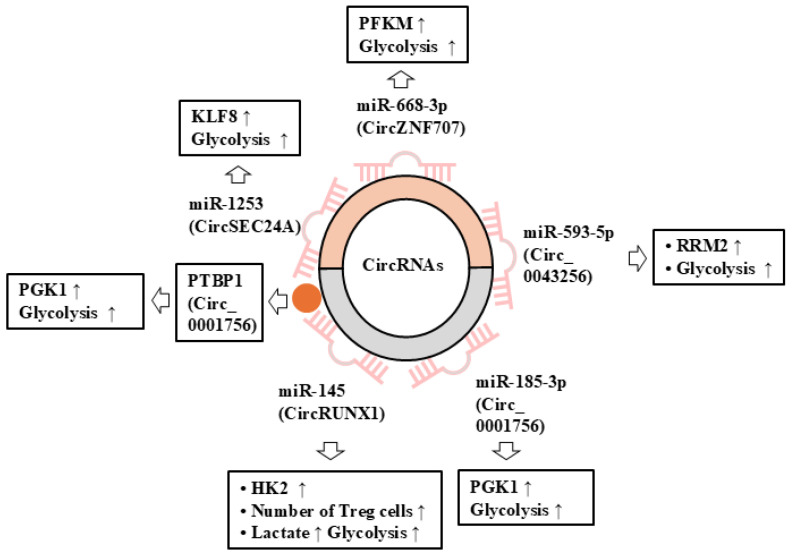
Roles of circRNAs in glycolysis. CircRNAs can sponge miRNAs to enhance glycolytic activity. Hollow arrows denote the direction of reaction. ↑ denotes the increased expression/activity.

Table 3 summarizes the roles of circRNA in glycolysis. Figure 5 shows the mechanisms of the regulation of glycolytic activity by circRNAs.

Since circRNAs can regulate both glycolysis and the response to ICIs, it is probable that glycolysis may affect the responses to ICIs. The inhibition of hexokinase 2 (HK2) decreases the number of MDSCs and enhances the efficacy of the ant-PD-L1 antibody in a tumor mouse model [108]. The glycolysis-associated gene signature displays potential as a high predictive marker of responsiveness to chemotherapy and immunotherapy in bladder cancers [109].

Treg cells display enriched glycolytic activity and high-level expression of immune checkpoint molecules such as TIGIT and CTLA-4 [110]. Increased glycolytic activity mediates anticancer drug resistance [111,112]. Glycolytic activity increases the expression of PD-1 in NSCLCs [113]. Oleanolic acid (OA) inhibits miR-130b-3p-induced glycolysis [114]. OA enhances the efficacy of the anti-PD-1 antibody by increasing the number of CD8^+^ T cells in HCC cells [114]. IGF2BP1 facilitates aerobic glycolysis and represses the function of CD8^+^ T cells in HCC [115]. IGF2BP1 enhances the stability of c-Myc mRNA, which in turn leads to the upregulation of PD-L1 [115]. Thus, targeting glycolysis can enhance the efficacy of anti-PD-1 immune therapy. Protein arginine methyltransferase 3 (PRMT3) promotes the progression of HCC [116]. PRMT3 promotes the production of lactate and inactivates the function of CD8^+^ T cells by increasing the expression of PD-L1 [116]. Thus, the PRMT3-lactate-PD-L1 axis can serve as a target for developing anticancer immunotherapy. The glycosylation of serine 249 (S249) on enolase 1 (ENO1) leads to the stabilization of PD-L1 by inhibiting the association of PD-L1 with ubiquitin E3 ligase STIP1 homology and U-Box-containing protein 1 (STUB1) [117]. Blockade of S249 glycosylation on ENO1 decreases the expression of PD-L1 expression and enhances anticancer immunity against colorectal cancer [117]. Thus, O-GlcNAcylation bridges aerobic glycolysis and immune evasion to suppress anticancer immunity.

These reports suggest that glycolytic pathways can be targeted for improving the response to anticancer immunotherapy. These reports also suggest that circRNAs can enhance the responses to ICIs by regulating the glycolytic pathway.

## 7. CircRNAs as Diagnostic and Prognostic Markers

Dysregulated expressions of circRNAs have been shown to be related to cancer progression and the response to anticancer therapy [118,119]. Many circRNAs are elevated in the sera and body fluids of cancer patients [69,120,121]. CircRNAs can be released from cells via extracellular vesicles [122,123]. CircRNAs typically exhibit tissue-specific and cell-specific expression patterns, implying their potential as biomarkers for disease diagnosis and prognosis [120,123,124]. The circRNAs show resistance to exonuclease activity, suggesting their roles as promising biomarkers for liquid biopsy in cancer detection and potential therapeutic targets for cancer treatment.

Hsa_circ_0000195 (circTTC13) is increased in HCC tissues [125]. The overexpression of circTTC13 is positively associated with poor prognosis and a low level of sorafenib-induced ferroptosis [125]. CircTTC13 sponges miR-513a-5p expression and increases the expression of SLC7A11, a negative regulator of ferroptosis [125].

Circ_0038632, highly expressed in breast cancer tissues [42], sponges miR-4306 to increase the expression of CXCR4 in breast cancer cells [42]. The downregulation of circ_0038632 suppresses the tumorigenic potential of breast cancer cells [42].

Circ_0015278 is decreased in lung adenocarcinomas [126] and suppresses tumor growth in mice by promoting ferroptosis through sponging miR-1228 [126].

Circular conformation and sequence overlap with linear cognate mRNAs have made the detection and quantification of circRNAs challenging. With the advent of new technologies, low-abundance circRNAs can be easily detected. Thus, circRNAs can be employed as diagnostic and prognostic markers. Machine learning and artificial intelligence will be helpful for the identification of more exosomal circRNAs. In the future, there will be expanded applications of circRNAs in cancer detection/prognosis and cancer therapeutics.

## 8. Synthesis of CircRNAs

CircRNAs have long been considered non-coding RNAs seemingly devoid of protein-coding potential. However, recent studies show the presence of protein-encoding circRNAs [127]. The presence of protein-coding circRNAs indicates that circRNAs can be employed for developing anticancer therapeutics. Although a majority of circRNAs are non-coding, artificial circRNA with internal ribosome entry sites (IRESs) can be translated to make protein. In vitro synthesized circRNAs can continue to translate into proteins for a longer time compared with linear mRNA vaccines [107]. The production of circRNA requires promoters, terminators, coding regions for antigens, untranslated regions (UTRs), IRES, RNA spacers, and homology arms. Homology arms and rationally designed spacers can increase the efficiency of circularization. The inclusion of poly(A) can enhance the translation of circRNA [128]. UTR provides a binding site for poly(A)-binding protein (PABP). The codon optimization of the coding region can enhance the translation of circRNAs. The translation of circRNA is mediated by the internal ribosomal entry site (IRES) or m6A-induced ribosome engagement site (MIRES) instead of 5′cap-dependent translation initiation [127]. The choice of IRES can affect translation efficiency [129]. The IRES of circRNA is recognized by eIF4G2 [130], and m6A modification is recognized by YTHDF3 [131] for its translation. The in vivo synthesis of circRNAs employs a vector (plasmid) that contains sequences that are circularized following transcription [132]. The in vivo synthesis of circRNAs requires an exonic region that is circularized and flanking intronic sequences [132]. The in vivo production system often results in the production of both circular and linear forms of RNA. In this review, we focus on the synthesis of circRNAs in vitro.

**Figure 6:**
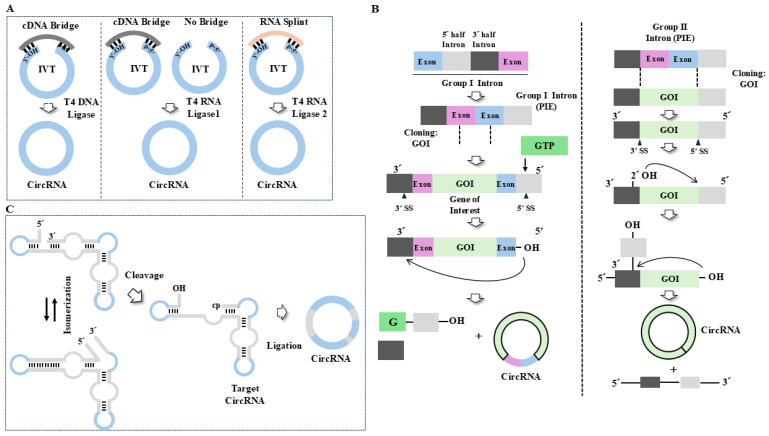
Circular RNA synthesis. (**A**) In vitro transcription produces linear RNA precursor, which is then ligated to yield circular RNA. T4 DNA ligase can yield circRNA with the aid of cDNA bridge. T4 RNA ligase 1 catalyzes the formation of a covalent 5′,3′-phosphodiester bond to produce circRNA. T4 RNA ligase 1 catalyzes nucleophilic attack of 3′ OH on the 5′ phosphate. The cDNA bridge is necessary for the linear RNA precursor to maintain stable structure. With the help of RNA splint, T4 RNA ligase 2 can produce circRNA with the help of RNA splint. T4 RNA ligase 2 is used when linear RNA precursor displays secondary structure with the ligation junction in the double-stranded region. IVT denotes in vitro transcription. Hollow arrows denote the direction of reaction. (**B**) CircRNA produced from group I self-splicing intron can yield circRNA containing exogenous exon sequences. In group II intron self-splicing reactions, exon sequences are dispensable. Group II intron self-splicing reactions lead to accurate ligation of linear RNA precursor. Ribozymatic circ RNA synthesis involves group I or group II intron-based permuted intron-exon (PIE) system. Hollow arrows and arrows denote the direction of reaction. 3′ and 5′ denote intron half. SS denotes splicing site. Dashed line denotes the site of cloning of gene of interest. (**C**) HPR method produces circRNA via rolling circle reaction and self-splicing reaction. The HPR is present in the circRNA. Hollow arrows denote the direction of reaction. Blue area denotes the target circRNA. Cp denotes 2′,3′-cyclic phosphate.

There are several methods for the synthesis of circRNA in vitro, such as the chemical method [133], enzymatic method [134] (Figure 6A), and ribozyme method [135,136] (Figure 6B,C). The production of circRNA is less complex, as it eliminates the need for nucleotide modifications, capping, and poly(A) tailing. The chemical method using cyanogen bromide (CNBr) or 1-Ethyl-3-(3-dimethylaminopropyl)carbodiimide (EDC) produces short circRNAs (50~70 nucleotides). Enzymatic methods employ T4 DNA ligase, RNA ligase 1, or RNA ligase 2 (Figure 6A). The T4 DNA ligase reaction employs a cDNA bridge and has shown inefficient ligation of in vitro transcribed RNA and DNA bridges. T4 RNA ligase 1 catalyzes the formation of covalent 3′,5′-phosphodiester bonds (Figure 6A). The cDNA bridge can also be used along with T4 RNA ligase 1 (Figure 6A). T4 RNA ligase 2 is suitable for linear RNA precursors containing a ligation junction in the double-stranded region. T4 RNA ligase 2 can also be used along with an RNA splint (Figure 6A). The introduction of a mutation can improve the binding efficiency of these enzymes to linear RNA and the production of selective circRNAs. These enzymatic reactions usually do not produce large circRNAs. These enzymatic reactions can induce intermolecular end-joining side reactions [137].

Recent advancements in group I and IIB self-splicing intron-based ribozymes have enabled the precise cyclization of RNA molecules [138]. The permuted intron–exon (PIE) system is employed for the expression of circRNAs, both in vitro and in vivo. The PIE method can be used for the synthesis of large circRNAs. This method eliminates the need for using enzymes. The PIE system employs a group I self-splicing reaction, which requires GTP and Mg^2+^. The RNA segment to be circularized is flanked by two halves of a split group I intron (Figure 6B). The PIE system contains exon sequences that are flanked by group I or group II intron sequences. Introns employed in the PIE system include *Anabaena* pre-tRNA introns, *Tetrahymena* group I introns, *Clostridium tetani* group II introns, and phage T4 td-encoded RNAs (Figure 6B). The PIE method can produce circRNA with high efficiency and precision. Since the PIE method employs exogenous exon sequences, the synthesized circRNA can be different from the original circRNA sequences [139]. These exogenous sequences may trigger unwanted immune responses [140]. The PIE method may not be useful for the synthesis of longer circRNAs. Compared to group I self-splicing intron reactions, group II self-splicing reactions involve reverse (back) splicing (Figure 6B). Group II self-splicing reactions can produce accurate circRNAs by joining the 5′ and 3′ ends of an exon (Figure 6B). Group II allows it to splice itself out of a precursor RNA molecule, leaving behind exons (Figure 6B). CircRNAs produced by a group II intron do not contain exogenous sequences (Figure 6B). RBPs can be useful for producing circRNAs without exogenous sequences [6]. However, this method leads to 2′,5′-phosphodiester bonds.

The hairpin ribozyme (HPR) method produces circRNAs from a single-stranded circular DNA template via a rolling circle reaction and natural self-splicing (Figure 6C). A circ DNA template containing HRP produces long-concatenated RNA molecules. The linear RNA precursor exists as two alternative conformations that are active in cleaving the 5′ and 3′ ends (Figure 6C). Linear monomeric HPR can cleave and join target RNAs in trans to produce circRNA. The HPR method is useful for producing small circRNAs. The disadvantage of this method is that circRNA may contain HPR sequences and produce unstable circRNA.

## 9. Roles of CircRNA Vaccines in Anticancer Immunity

**Figure 7:**
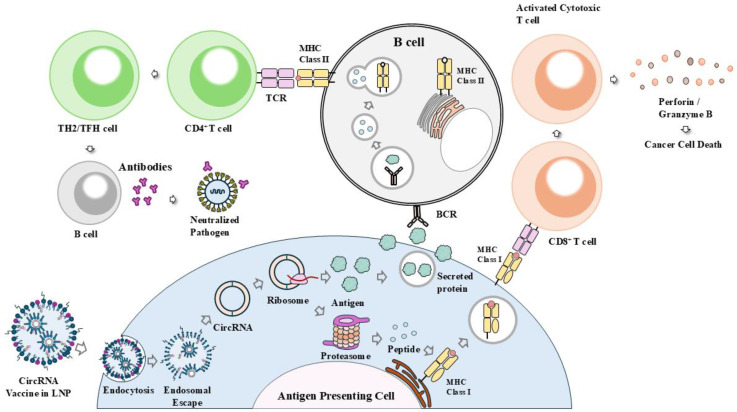
Vaccines employing circRNAs induce cell-mediated and humoral immunity. CircRNA-LNP vaccine is endocytosed by antigen presenting cells. Following endosomal escape, CircRNAs are then translated into corresponding proteins. Proteins undergo proteasomal degradation. Peptides are then presented on MHC-I to induce activation of CD8^+^ T cells. Activated CD8^+^ T cells kill tumor cells by granzyme B (GzmB) and perforin. Secreted proteins are recognized and engulfed by antigen presenting cells such as B cells. Antigenic peptides are presented on the MHC-II of B cells to induce activation of CD4^+^ T cells. Activated CD4^+^ T cells, such as TH2 cells and TFH cells, can activate B cells to induce production of antigen-specific antibodies. Hollow arrows denote the direction of reaction. TH2 denotes T helper 2 cells and TFH cells denote T follicular helper cells. LNP denotes the lipid nanoparticle. BCR denotes the B cell receptor. TCR denotes the T cell receptor.

CircRNAs can be templates for translation. CircRNAs can be translated with m6A modifications or IRES. CircRNAs can encode proteins after entering the cytoplasm and do not cause insertional mutagenesis, which could offer new possibilities for therapeutic applications (Figure 7). Due to high stability and translation efficiency, circRNAs can be employed as cancer therapeutic vaccines. Compared to linear mRNA vaccines, circRNAs display low immunogenicity, even without a base modification [10]. Thus, circRNAs can serve as candidates for anticancer vaccine development. The circRNA can be administered directly in a naked form or encapsulated in delivery carriers, such as lipid nanoparticles (LNPs).

The circRNA vaccine with internal ribosome entry sites (IRESs) and an open reading frame (ORF) provides an improved approach to linear mRNA-based vaccination with safety, stability, and scalability [141,142].

CircRNAs developed by using group IIC self-splicing introns can produce exogenous proteins and induce strong immune responses against respiratory syncytial virus [135]. The intranasal circRNA vaccine encapsulated in LNP (D2GFP) can produce antigenic peptides and induces strong antitumor T-cell responses in lung cancer models without displaying systemic immune adverse effects [143]. CircRNA in LNP can be efficiently delivered to dendritic cells in draining lymph nodes to induce antigen-specific T-cell responses in mice [144]. CircRNA-encoding protein antigen induces the activation of dendritic cells, antigen-specific CD8^+^ T-cell responses in lymph nodes and tissues in a mouse model [145] (Figure 7). CircRNA vaccines can protect macaques against variants of SARS-CoV-2, with higher efficacy than linear mRNA vaccines [146]. The circRNA vaccine in LNP produces more stable antigen production than the 1mΨ-modified mRNA vaccine [128,146]. The circRNA-LNP exhibits greater thermostability than linear mRNA-LNP vaccines [10]. CircRNA-LNP induces robust innate and adaptive immune response in three tumor models [147].

Anticancer vaccines target tumor-associated antigens (TAAs) and tumor-specific antigens (TSAs) and eliminate cancer cells. There have been reports concerning the roles of circRNA vaccines encoding TAAs or TSAs in anticancer immune responses. CircMYH9 encodes tumor neoantigen and eliminates tumor-derived organoids by activating the function of CD8^+^ T cells [148]. CircRNA targeting H19 immune-related protein (H19-IRP) induces cytotoxic T-lymphocyte (CTL) responses and suppresses glioblastoma (GBM) growth [149]. CircRNA encapsulated in LNP suppresses the growth of pan-adenocarcinoma xenografts [122,150]. The circRNA-loaded DC vaccine, in combination with gemcitabine, displays a tumor inhibition rate of 89% in a pancreatic cancer model. The combined treatment reduces the number of Tregs and suppresses immune evasion [151].

Small circRNAs are usually produced through the hairpin ribozyme method via rolling circle replication [152]. Small circRNA vaccines encoding TAAs, TSAs, or viral antigens elicit robust CD8^+^ and CD4^+^ T-cell responses in young adult mice and in immunosenescent aged mice [55]. Antigen-encoding small circRNAs (circRNAs) loaded in LNP induce robust antitumor immunity against mouse melanoma when combined with immune checkpoint inhibitors [55].

The intratumoral delivery of circRNA encoding a mixture of cytokines induces strong antitumor immune responses and enhances anti-programmed cell death protein 1 (PD-1) antibody-induced tumor regression in a syngeneic mouse model [153]. Thus, cytokine-encoding circRNA vaccines can be employed as adjuvants in immune therapy employing immune checkpoint inhibitors.

The utilization of PD-1/PD-L1 inhibitors contributes to the advancement of cancer immune therapy. The increased expression of PD-L1 can cause immune evasion. By modulating PD-L1 levels, circRNAs can enhance the efficacy of anticancer immunotherapies. CircCDYL activates mTORC1-p70S6K signaling by stabilizing the hornerin (HRNR) protein [154] and increases the expression of PD-L1 and promotes stemness in hepatocellular carcinoma cells [154]. CircRNA vaccines in combination with immune checkpoint inhibitors can enhance anticancer immunity. LNP-encapsulated cEMSY in combination with anti-PD-1 antibody induces a potent antitumor immune response in an immunosuppressed tumor model without apparent toxicities [55]. Circ_0001947 is increased in GC tissues and can predict poor prognosis in GC patients [155]. Circ_0001947 in small extracellular vesicles (sEVs) promotes CD8^+^ T-cell exhaustion by sponging miR-661 and miR-671-5p [155]. The blockade of circ_0001947 by small hairpin RNA (shRNA) enhances the sensitivity of GC cells to anti-PD-1 therapy [155]. The combination of inhibitor of circ_0001947 with ICIs may offer hope for more effective and personalized treatments. It will be necessary to identify more circRNAs that can predict the responses to ICIs for developing effective circRNA vaccines. Figure 7 shows that the translated circRNA vaccine activates both CD4^+^ T-cell and CD8^+^ T-cell immune responses.

## 10. Clinical Trials of CircRNA Vaccines and CircRNAs

Table 4 lists clinical trials involving circRNA vaccines. A clinical trial (NCT06530082) aims to (1) determine the safety and tolerability of CircFAM53B-219aa DC vaccine monotherapy; (2) determine the efficacy of a combination of the CircFAM53B-219aa DC vaccine with camrelizumab in the treatment of HER2-negative advanced breast cancer. The study results have not been posted. CircFAM53B encodes HLA-A*02:01-restricted peptide CircFAM53B-219aa and induces antigen-specific T-cell responses. CircFAM53B-219aa-loaded DC cells can activate antigen-specific T cells and can suppress the growth of tumors with high CircFAM53B expression [156].

Clinical trial (NCT04584996) aims to (1) define the circRNA expression profile in pancreatic ductal adenocarcinoma (PDAC); (2) evaluate candidate circRNA expression in blood (plasma samples); (3) determine the expression of candidate circRNAs and related molecules in patient biomaterials (tissue, blood, and biopsy samples); (4) describe the theoretical interactions of candidate circRNAs within the full complement of RNA and related molecules (transcriptome analysis). No results have been posted.

Clinical trial (NCT06042842) aims to (1) evaluate the clinical relevance of plasma circRNAs (hsa_circ_0004001) as a noninvasive diagnostic biomarker for HCC; (2) determine the relation of circRNAs (hsa_circ_0004001) to HCC staging; (3) compare between circRNAs (hsa_circ_0004001) and the routine marker (AFP) as biomarkers for HCC diagnosis. The study results have not been submitted.

Clinical trial (NCT05934045) aims to determine whether circRNAs can be used as prognostic and/or predictive biomarkers of Anaplastic lymphoma kinase (ALK) + Anaplastic large-cell lymphoma (ALCL) resistance to treatment. The study results have not been submitted.

Clinical trial (NCT06617585) aims to study the role of circDENND4c in the diagnosis of ovarian cancer. The study results have not been submitted.

More clinical trials will also validate the potential of circRNAs as diagnostic makers and targets for developing anticancer therapeutics. The results of clinical trials on circRNAs on LNPs have not been reported. Further clinical trials involving circRNA vaccines are necessary for improving the safety and efficacy of circRNA vaccines. Table 4 summarizes the clinical trials concerning combinations of the circRNA vaccine with chemotherapy and circRNAs as biomarkers.

## 11. Discussion and Perspectives

CircRNAs play important roles in cancer progression, metabolism, gene editing [157], resistance to anticancer drugs and ICIs. The diverse functional roles of circRNAs make them valuable targets for developing anticancer therapeutics. CircRNAs compensate for the shortcomings of linear mRNAs and have the potential to become a reliable vaccine candidate. The circularization of SiRNA mimic [158], RNA aptamers [159], or RNA dumbbells [160] may improve their stability and efficacy. The development of circRNA vaccines is still at a nascent stage, with very few reports on their in vivo studies and clinical trials.

CircRNA vaccines must meet several requirements to be effective therapeutic options. Since circRNAs perform various biological functions, in vitro synthesized circRNAs may cross react with their latent interactions, resulting in unwanted functions or even serious side effects. Therefore, the sequences of artificial circRNAs should be specific and are adequately tested for the induction of unwanted immunity and efficacy in preclinical evaluations. Chemical and enzymatic methods may cause low efficiency of the circularization of linear RNA precursors. This low efficiency leads to unwanted immune responses. An intermolecular end-joining reaction occurs during circRNA synthesis. It is, thus, necessary to optimize the concentration of the linear RNA precursor to limit end-joining reactions. CircRNA vaccines may contain residual linear precursor mRNAs, which may cause immune adverse reactions by activating innate immunity. Thus, the complete purification of circRNA is necessary for minimizing side reactions. A combination of gel electrophoresis, RNase R degradation [161], gel filtration, and ultrafiltration can be used for the large-scale purification of circRNAs. Since the structure of the linear RNA precursor determines circRNA synthesis yield, the addition of unnatural nucleotides during the synthesis of linear RNA precursors may improve the efficiency of ligation. The PIE system containing a group I intron produces circRNA containing exogenous sequences (scar sequences), which can induce unwanted immune responses. It is necessary to modify the PIE construct to produce circRNAs without scar sequences. The RNA duplex formed during circRNA synthesis can activate the degradation of circRNA by PKR [162]. It is, therefore, important to minimize the formation of the RNA duplex. The optimization of reaction conditions and reaction components may increase the production of circRNAs.

Negative charges and large molecular sizes of cirRNAs require suitable systems for their delivery. Delivery vehicles include LNPs [144], gold nanoparticles [163], adeno-associated viral vectors (AAVs) [164], and lentiviral vectors [165]. The inducible lentiviral vector system displays accuracy and reliable expression of circRNA in various cell types [165]. CircRNA in LNP can efficiently induce immune responses and display minimal immune adverse events [166]. Targeted delivery of circRNA vaccines is crucial for enhanced therapeutic effects and minimal off-target effects. To prevent liver injury, it is necessary to develop organ-specific delivery of circRNA in LNP. Mannose-modified LNPs make it possible for delivery into lymph nodes and induce potent and persistent immune responses [144]. Although LNPs are the most widely employed delivery system, it is necessary to increase the loading capacity and stability of LNPs. LNPs mostly contain polyethylene glycol (PEG), and PEG displays immunogenicity [167]. COVID-19 mRNA-LNP vaccines (Comirnaty and Spikevax) induce allergic reactions by increasing the levels of anti-PEG IgG/IgM [168]. Anti-PEG IgG/IgM may decrease the efficacy of circRNA vaccines by causing the accelerated blood clearance (ABC) phenomenon. Thus, circRNA vaccines employing PEGylated LNP may cause allergic reactions. It will be necessary to replace PEG with other polymers. AAVs show minimal pathogenicity and long-term gene expression [169]. AAVs can deliver DNA that encodes circRNA to achieve efficient circRNA synthesis [170]. Extracellular vesicles containing circRNAs are not likely to be rejected, making them a reasonable delivery vehicle for circRNAs [171]. Thus, exosomes can replace LNP as a delivery vehicle for circRNAs. Further clinical trials and research into these delivery vehicles will be necessary for developing safe, stable, and efficient circRNA vaccines.

Since conventional tumor-associated antigen TAAs usually display low immunogenicity [172], vaccines employing TAAs may not be effective against cancer patients expressing TAAs. Thus, the identification of novel TSAs is necessary for developing effective cancer vaccines. Tumor-specific neoantigens induce strong immune responses by activating cytotoxic type 1 regulatory T (Tr1) cells [173]. The application of artificial intelligence (AI) can predict and identify immunogenic neoantigens [174]. These discoveries of novel tumor antigens will facilitate the development of circRNA-based vaccines. The optimized tumor-specific antigen circRNA vaccine platform can be a stable, efficient, and reliable alternative platform to conventional mRNA vaccines for cancer immunotherapy. Further clinical trials will make it possible for the widespread use of circRNA vaccines for preventing and treating tumor malignancies. So far, no circRNA vaccines have received approval for use as anticancer drugs.

Combining the RNA vaccine with CAR-T cells targeting tumor antigen claudin 6 (CLD6) can enhance the efficacy of CAR-T cells in relapsed solid tumors [175]. Chimeric antigen receptor-T cells (CAR-T) based on circRNA encoding the anti-Delta-like Ligand 3 (DLL3) show enhanced efficacy toward small cell lung cancer (SCLC) compared to CAR-T cells based on mRNA [176]. Thus, circRNAs can enhance the efficacy of CAR-T cells. Immune checkpoint inhibitors have shown potential as cancer therapeutics. However, resistance to these inhibitors eventually develops. A combination of the circRNA vaccine with immune checkpoint inhibitors or CAR-T cells may enhance adaptive immune responses. Clinical trials involving circRNAs and ICIs or CAR-T cells may lead to improvements in the design of safe and efficient circRNA vaccines.

It is known that circRNAs can confer resistance to chemotherapy and ICIs. Uncovering the mechanisms of circRNA-mediated therapeutic resistance in detail could identify new therapeutic targets or biomarkers, enabling precise personalized therapy. Results from clinical trials of the circRNA vaccine to overcome resistance to anticancer therapeutics have not been reported.

Since circRNAs can act as an oncogene [125] or tumor suppressor [126], it is reasonable that endogenous circRNAs can serve as targets for developing anticancer therapeutics. Targeting circRNAs involves RNA interference (RNA i) and the CRISPR/Cas 13 system [177]. The RNAi system targets back-splice junctions of circRNAs. The RNAi system employs short-interfering RNA or short hairpin RNA. This RNAi system does not affect the expression of linear RNAs. The CRISPR/Cas 13 system employs inactive Cas13 protein and guides RNA. The RNAi system often causes off-target effects. The downregulation of hsa_circRNA_0000585 by RNAi suppresses the tumorigenicity of ovarian cancer cells by inhibiting autophagic flux [178]. It is necessary to reduce the potential off-target effects associated with the RNAi system. Clinical trials of the RNAi system targeting endogenous circRNAs are needed for developing safe and efficacious anticancer therapeutics.

**Figure 8:**
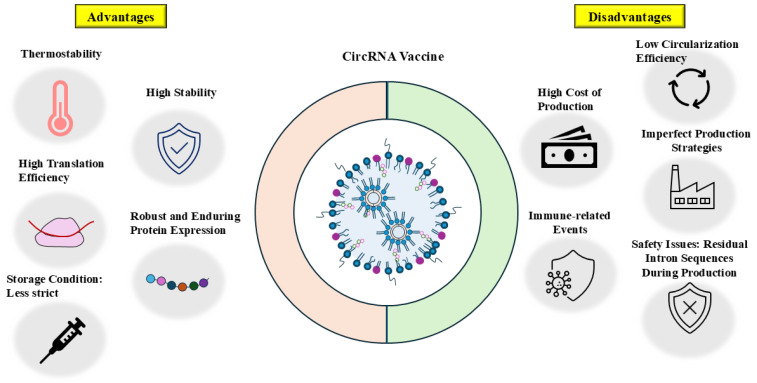
Advantages and disadvantages of circRNA vaccines.

The advantages of circRNAs will make them an alternative to conventional mRNA vaccines. CircRNAs have shown potential in preclinical models and will play important roles in medical applications. Clinical trials concerning the efficacy of circRNA vaccines along or combined with ICIs or chemotherapy drugs are necessary for improving the safety and efficacy of circRNA vaccines. Figure 8 shows the advantages and disadvantages of circRNA vaccines.

## Figures and Tables

**Table 1:** Roles and mechanisms of circRNAs in anticancer drug resistance.

Circular RNAs	Effect on Drug Resistance	Mechanism	Refs
CircCCNY	Sensitivity to lenvatinib ↑	Degradation of HSP60 Interaction with E3 ubiquitin ligase SMURF1	[54]
Circ_0000376	Docetaxel resistance ↑	Sponging miR-1298-5p	[59]
Circ_0076305	Paclitaxel resistance ↑	Sponging miR-936 TMPRSS4 ↑	[62]
CircQKI	Docetaxel resistance ↑	Sponging miR-188-3p	[63]
Circ_0058608	Taxol resistance ↑	Sponging miR-1299 GBP1 ↑	[64]
CircRNA_0044556	Paclitaxel resistance ↑	Sponging miR-936	[65]
Circ_0005667	Cisplatin resistance ↑	Sponging miR-145-5p IGF2BP1 ↑	[66]
CircKIAA0182	Cisplatin resistance ↑	Interaction with RBP YX1	[70]
CircFRMD4A	Sensitivity to elesclomol ↑	Inactivation of pyruvate kinase M2	[73]

↓ denotes the decreased expression/activity and ↑ denotes the increased expression/activity.

**Table 2:** Effects of circRNAs on immune response.

Circular RNAs	Effect on Immune Responses	Mechanism	Refs
CircPETH_147aa	Immune evasion	SLC43A2 mRNA stability ↑ Causes methionine deficiency Apoptosis of CD8^+^ T cells ↑	[77]
Circ_CPA4	Immune evasion	Sponging let-7 miRNA PD-L1 ↑	[78]
Circ_CHST15	Immune evasion	Inhibits CD8^+^ T cell activity PD-L1 ↑	[79]
CircENTPD7	Immune evasion	IGF2BP/PD-L1 ↑	[81]
CircFAM64A(3)	Immune evasion	Sponging miR-149-5p PD-L1 ↑	[82]
CircAATF	Immune evasion	Sponging miR-142-5p PD-L1 ↑ pAKT ↑	[83]
CircMGA	Immune activation	CD8^+^ T cell activity ↑ CCL5 ↑	[46]
Circ_001377	Immune evasion	Sponging miR-498-3p PD-L1 ↑ IFNGR2 ↑	[84]
CircDLG1	Immune evasion	Sponging miR-141-3p CXCL12	[86]
CircPHLPP2	Immune evasion	Binding to ILF3 Inhibits NK cell function	[87]
CircNCOA3	Immune activation	Number of CD8^+^ T cells ↑ Number of MDSCs ↓	[88]
CircFGFR4	Immune evasion	Sponging miR-185-5p CXCR4 ↑ Confers resistance anti PD-1	[90]
CircCFL1	Immune evasion	PD-L1 ↑ C-Myc ↑ CD8^+^ T cell activity ↓ Confers resistance to anti PD-1	[91]
CircNF1	Immune evasion	Activation of JAK-STAT3 PD-L1 ↑ Interaction between USP7 and PD-L1 ↑	[93]
CircRHBDD1	Immune evasion	IGF2BP2↑ Interaction between IGF2BP2 and TRIM25 ↓ Infiltration of CD8^+^ T cells ↓ PD-L1↑	[94]
CircSOD2	Immune evasion	Sponging miR-497-5p ANXA 11 ↑ Anti-PD-1 resistance	[96]
CircQSOX1	Immune evasion	Sponging miR-326 and miR-330-5p PGAM1 ↑ Anti-CTLA-4 resistance	[98]
Exosomal circCCAR1	Immune evasion	Sponging miR-125-5p PD-1 ↑ CD8^+^ T cell activity ↓	[100]
Exosomal circWDR25	Immune evasion	Sponging miR-4474-3p CTLA-4 ↑PD-L1 ↑	[101]
Exosomal circUHRF1	Immune evasion	Sponging miR-449c-5p TIM-3 ↑ Inhibition of NK cell function	[102]

↓ denotes the decreased expression/activity and ↑ denotes the increased expression/activity.

**Table 3:** Roles of circRNAs in glycolysis.

Circular RNAs	Effect on Glycolysis	Mechanism	Refs
CircPVT1	Enhances glycolytic activity	Glutaminolysis ↑ Binding to miR-33a-5p	[4]
circZNF707	Enhances glycolytic activity	PFKM ↑ Binding to miR-668-3p	[45]
CircRUNX1	Enhances glycolytic activity	HK2 ↑ Binding to miR-145	[95]
CircSEC24A	Enhances glycolytic activity	miR-1253 ↓ Krueppel-like factor 8 (KLF8) ↑	[104]
circST6GALNAC6	Inhibits glycolysis	HK1 degradation Parkin E3 Ubiquitin Ligase ↑	[105]
Circ_0043256	Enhances glycolytic activity	miR-593-5p ↓ RRM2 ↑	[106]
Circ_0001756	Enhances glycolytic activity	Binding to PTBP1, miR-185-3p PGK1 ↑	[107]

↓ denotes the decreased expression/activity and ↑ denotes the increased expression/activity.

**Table 4:** Clinical trials involving circRNAs and circRNA vaccines.

Title	Phase	Condition or Disease	Prospective Outcome Measures	Dates	ID/Purpose
A Single Arm Clinical Study of Dendritic Cell Vaccine Loaded With Circular RNA Encoding Cryptic Peptide for Patients With HER2-negative Advanced Breast Cancer	Phase I, not yet recruiting, enrollment 48	Advanced breast cancer	Biological: CircFam53B-219aa DC vaccine Drug: camrelizumab Incidence of Dose-Limiting Toxicity (DLT) Incidence of immune adverse events Objective Response Rate (ORR)/Overall Survival (OS)/Progression Free Survival (PFS)	1 December 2024 (start) 1 January 2027 (completion)	NCT06530082/Treatment
CIRcular and Non-coding RNAs as Clinically USeful Biomarkers in Pancreaticobiliary Cancers	Enrollment 186	Pancreatic cancer, Biliary tract cancer	Identify dysregulated circRNA candidate(s). Evaluate candidate circRNA expression in blood Explore the expression of candidate circRNAs, and related molecules	4 October 2020 (start) 5 November 2023 (completion)	NCT04584996/Observational
The Value of circRNAs (hsa_circ_0004001) in Early Diagnosis of HCC	Enrollment 186	Hepatocellular carcinoma	Evaluate the clinical utility of plasma circRNAs (hsa_circ_0004001) between circRNAs (hsa_circ_0004001) and the routine marker (AFP)	November 2023 (start) February 2025 (completion)	NCT06042842/Observational
Deciphering the Role of Circular RNAs in ALK positive Anaplastic Large-cell Lymphoma (CIRComa)	Active, not recruiting Enrollment 80	Anaplastic Large-cell Lymphoma	Number of participants with relapse and circulating circRNA identification of a signature of circRNAs associated with therapy resistance RNA seq.	1 January 2023 (start) 31 December 2025 (completion)	NCT05934045/observational
The Role of CircDENND4C in Epithelial Ovarian Cancer	Active, not recruiting Enrollment 30	Epithelial ovarian can cer	role of circDENND4c in the diagnosis of ovarian cancer real time PCR	1 December 2024 (start) 1 October 2028 (completion)	NCT06617585/observational

## Data Availability

No new data were created in this study.
